# Cricohyoidoepiglottopexy in an emergency scenario: Evaluation and management of a severe laryngeal trauma

**DOI:** 10.1002/ccr3.6304

**Published:** 2022-10-06

**Authors:** Andrea Di Laora, Anne‐Laure Capitaine, Mathilde Lacour, Jean‐Paul Trijolet

**Affiliations:** ^1^ Department of ENT and Head & Neck Surgery Groupe Hospitalier Littoral Atlantique La Rochelle France; ^2^ Department of Radiology Groupe Hospitalier Littoral Atlantique La Rochelle France

**Keywords:** cricohyoidoepiglottopexy, laryngeal fracture, neck trauma

## Abstract

We describe the clinical evaluation and the management of a severe laryngeal trauma in a suicidal patient. We aim to demonstrate how the cricohyoidoepiglottopexy, which is a surgical technique mainly performed for oncological purposes, can be successfully used in this emergency setting.

## Graphical abstract

We describe the clinical evaluation and the management of a severe laryngeal trauma in a suicidal patient. We aim to demonstrate how the Cricohyoidoepiglottopexy, which is a surgical technique mainly performed for oncological purposes, can be successfully used in in this emergency setting.

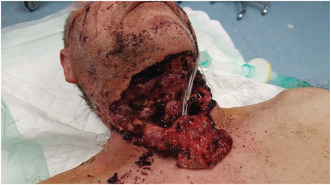

## 1 INTRODUCTION

Laryngeal fracture is an infrequent acute traumatic injury in the neck area. Once a laryngeal fracture is suspected or identified, the primary goal is to secure the airway, followed by reconstruction to preserve laryngopharyngeal function. We describe the case of a laryngeal trauma whose emergency treatment required the technique of cricohyoidoepiglottopexy (CHEP).

## 2 CASE REPORT

A 71‐year‐old man, with no significant medical history, attempted autolysis by severing his neck with a circular saw. He was urgently transferred to our intensive care unit, and he was intubated through the cervical wound at the level of the laryngeal opening.

**FIGURE 1:**
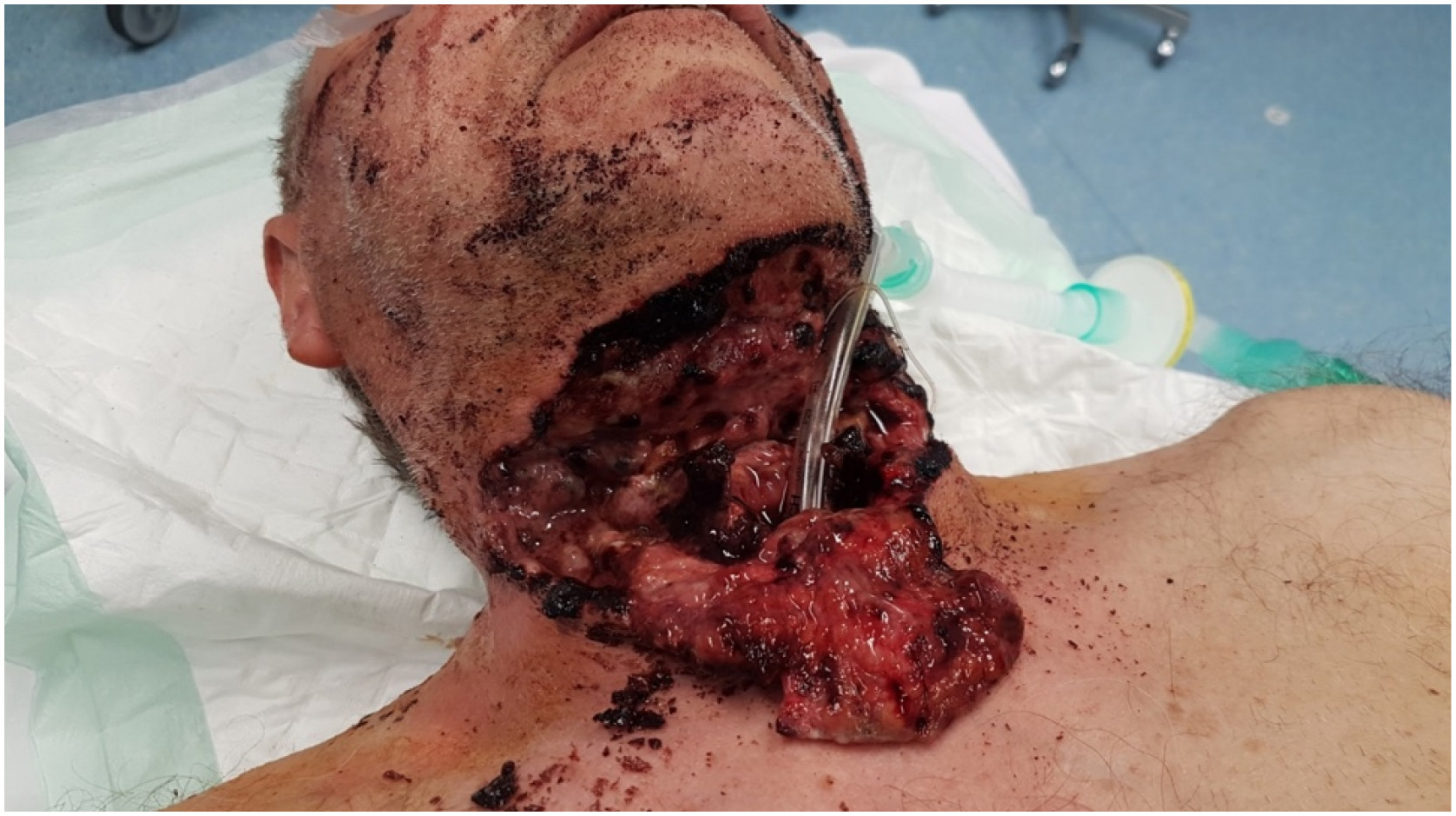
Patient presentation at the emergency services, the intubation was performed through the cervical wound at the level of the laryngeal opening.

Physical examination revealed a cervical wound extending from each sternocleidomastoid muscle, forming a cutaneous hinge flap (Figure 1).

**FIGURE 2:**
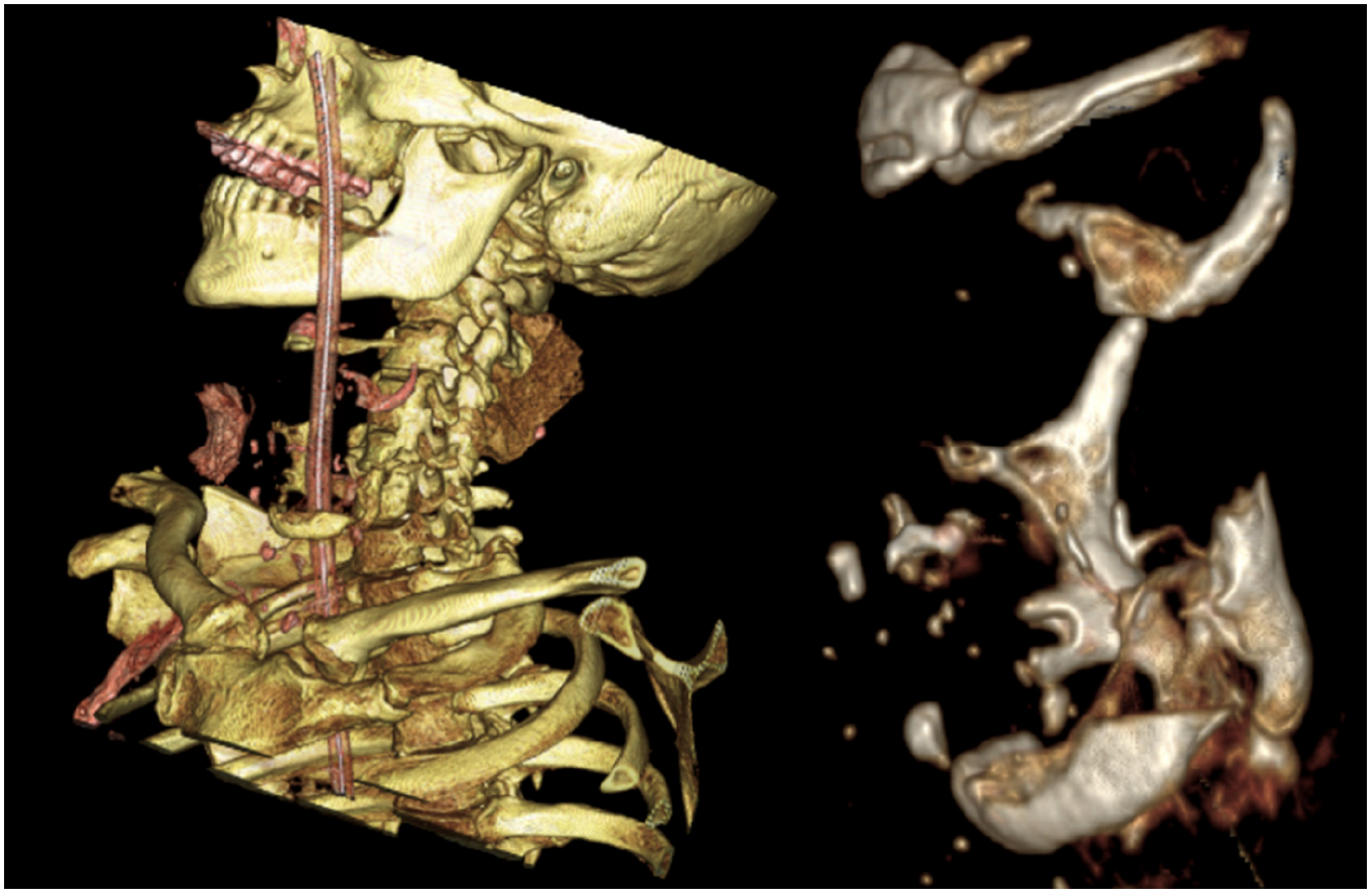
Three‐dimensional CT scan reconstructions of the neck (left) and of the larynx (right), showing a double fracture of thyroid cartilage with an absence of the anterior part of the scutum. The reconstructions were made from the available axial standard images in the PACS system.

A computerized tomography (CT) of the neck and thorax was performed. Hyoid bone and cricoid cartilage were intact. The three‐dimensional reconstructions showed a double fracture of thyroid cartilage with an absence of the anterior part of the scutum and of vocal folds (Figure 2), consistent with a Schaefer type 4 laryngeal injury (Table 1).

**TABLE 1:** Patient presented a laryngeal trauma of severity IV according to the Schaefer Fuhrman's classification

Severity of laryngeal injury (Schaefer Fuhrman's classification)	
Group	Injury
I	Minor endolaryngeal hematoma without detectable fracture
II	Edema, hematoma, minor mucosal disruption without exposed cartilage, and nondisplaced fractures
III	Massive edema, mucosal disruption, exposed cartilage, vocal fold immobility, and displaced fracture
IV	Group with disruption of anterior larynx, unstable fractures, two or more fracture lines, or massive trauma to laryngeal mucosa
V	Complete laryngotracheal separation

Urgent surgery was performed. The primary step was to secure the upper airways by performing a tracheotomy under the thyroid gland isthmus, between the third and fourth tracheal ring, with a cutaneous access separated from the cervical wound. A flexible endotracheal tube Montandon Vygon caliber 8 mm was put in place. After disinfection of the cervical wound, a debridement of necrotic strap muscles was done. On neck exploration, the pre‐epiglottic space was found to be lacerated with resection of the epiglottis, of the anterior part of the thyroid cartilage. The hyoid bone and the supra hyoid epiglottis were intact, no trauma of the trachea was found. The posterior edges of the thyroid cartilage had been skeletonized and removed leaving the inferior and superior horns to protect the recurrent nerves and the uppers peduncles, respectively. The anatomy of the two cricoarytenoid joints was respected but a right arytenoid subluxation was present. The large defect of the thyroid cartilage prevented the possibility of repairing the anterior commissure, and we decided to perform a supracricoid horizontal partial laryngectomy with CHEP to restore the laryngopharyngeal function. Each residual vocal fold, anchored to the vocal process of the arytenoid, was resected to avoid a glottic stenosis. The pexy was performed by three stitches with an absorbable suture thread size n°2 and a large needle (48 mm).

**FIGURE 3:**
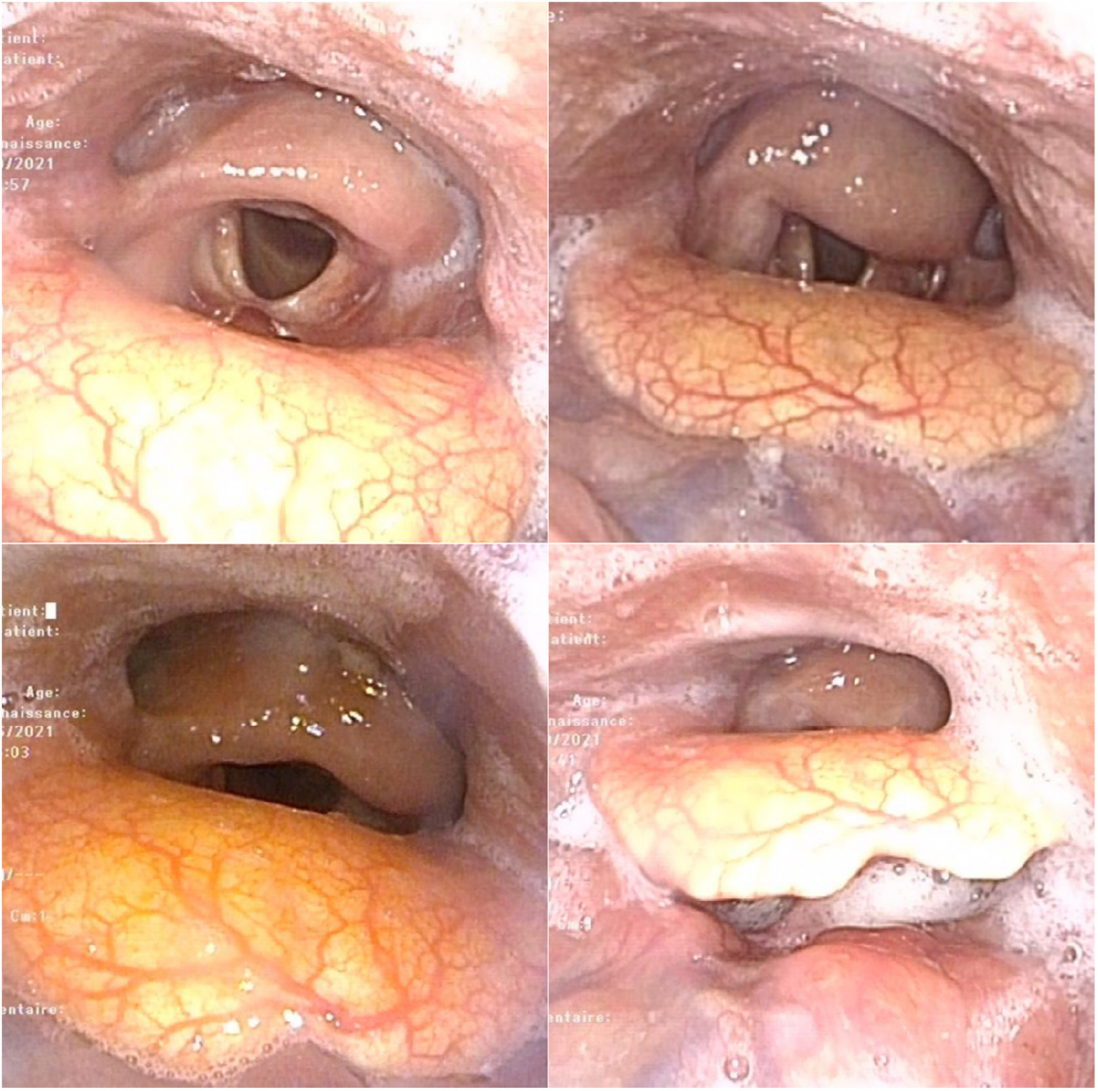
Images of the flexible endoscopy examination on the twentieth day showing the postoperative anatomical and functional outcomes. The epiglottis perfectly covers the glottidis during swallowing.

The postoperative course had no complications. On the third day, the cap of the cannula was deflated and a phonatory valve was put in place. The flexible endoscopy examination showed a mobile left arytenoid, an opened left pyriform sinus, the right cricoarytenoid joint was not functional (Figure 3). Speech and swallow rehabilitation were started early. Feeding was ensured by a nasogastric tube during the first 15 days and then by a gastrostomic probe to facilitate the rehabilitation of swallowing and to ensure sufficient nutritional intake. On the twentieth day, the patient was decannulated and the patient did not complain of dyspnea. The diet included thickened water, homogeneous foods with creamy structure. After 55 days, the patient had no dysphagia, no pulmonary complications, and the fiber endoscopic evaluation of swallowing showed no signs of food or drink inhalation. The nutritional assessment was normal, and the removal of the gastrostomic probe was carried out. The patient had a strict psychiatric follow‐up and to the present situation he has had no suicidal ideation.

## 3 DISCUSSION

Laryngeal injuries are rare accounting for less than 1% of all trauma.^1^ The mean etiology of an external laryngeal trauma is a blunt or penetrating trauma. Neck wounds that extend deep into the platysma are considered penetrating trauma and comprise 5%–10% of all trauma cases that present to the emergency department.^2^ Our paper describes a case of penetrating trauma of the neck resulting from an unusual process of attempting autolysis by means of a circular saw.

The first goal in patients with laryngeal trauma is to establish a secure airway. Orotracheal intubation is not recommended because of causing further damage to the larynx. For our patient, intubation was performed directly by the cervical wound and the larynx opening.

The next goal is to restore anatomic relationships to enable return of laryngopharyngeal function. Laryngeal reconstruction should be performed within the first 24 to 48 h after a traumatic fracture^3^ to prevent infection, tissue necrosis, and bare mucous areas. In our case, the laryngeal injuries involved a complete resection of the pre‐epiglottic space, of the thyroid cartilage and of the vocal cords up to the upper edge of the cricoid. For this reason, we decided to restore the larynx using a reconstructive laryngectomy according to Mayer and Piquet surgical technique.^4^ A nononcological use of this technique to restore laryngeal function in a case of laryngeal trauma was also reported by Ferreira.^5^ Due to the damage to the thyroid cartilage, they considered the CHEP as the simplest, safest, and most effective way to restore laryngeal function.

## 4 CONCLUSION

Laryngeal penetrating trauma is uncommon and life threatening with a long‐term management challenge. We recommend securing the airway and a primary closure. CHEP is a surgical approach used for oncological purposes in the management of glottic carcinoma but, as this case shows, this surgical technique must be a part of the surgeon's knowledge in particular emergency settings.

## AUTHOR CONTRIBUTIONS

DI LAORA A drafted, revised carefully, and approved the submitted version of the manuscript. CAPITAINE AL revised the manuscript drafts and approved the submitted version of the manuscript. LACOUR M provided the 3D CT scan images, revised the manuscript drafts, and approved the submitted version of the manuscript. TRIJOLET JP revised the manuscript drafts and approved the submitted version of the manuscript.

## CONFLICT OF INTEREST

The authors have no financial relationships or conflicts of interest to disclose.

## ETHICAL APPROVAL

This case report did not meet the criteria to require IRB approval.

## CONSENT

Written consent from the patient was obtained to publish this report in accordance with the journal's patient consent policy.

## Data Availability

Data sharing is not applicable to this article as no datasets were generated or analyzed during the current study.
